# Cyclically stretched ACL fibroblasts emigrating from spheroids adapt their cytoskeleton and ligament-related expression profile

**DOI:** 10.1007/s00441-021-03416-9

**Published:** 2021-04-09

**Authors:** Clemens Gögele, Christina Hoffmann, Jens Konrad, Rudolf Merkel, Silke Schwarz, Mersedeh Tohidnezhad, Bernd Hoffmann, Gundula Gesine Schulze-Tanzil

**Affiliations:** 1 Institute of Anatomy and Cell Biology, Paracelsus Medical University, Prof.-Ernst-Nathan Str. 1, 90419 Nuremberg and Salzburg, Nuremberg, Germany; 2 Department of Biosciences, Paris Lodron University Salzburg, Hellbrunnerstr. 34, 5020 Salzburg, Austria; 3 Institute of Biological Information Processing: IBI-2, Forschungszentrum Jülich, 52425 Jülich, Germany; 4 Department of Anatomy and Cell Biology, RWTH Aachen University, Wendlingweg 2, 52074 Aachen, Germany

**Keywords:** Cyclic strain, Uniaxial stretch, ACL-derived fibroblasts, Mechanostimulation, Spheroids, Tendon extracellular matrix, Mohawk, Myodulin, Connexin 43

## Abstract

Mechanical stress of ligaments varies; hence, ligament fibroblasts must adapt their expression profile to novel mechanomilieus to ensure tissue resilience. Activation of the mechanoreceptors leads to a specific signal transduction, the so-called mechanotransduction. However, with regard to their natural three-dimensional (3D) microenvironment cell reaction to mechanical stimuli during emigrating from a 3D spheroid culture is still unclear. This study aims to provide a deeper understanding of the reaction profile of anterior cruciate ligament (ACL)-derived fibroblasts exposed to cyclic uniaxial strain in two-dimensional (2D) monolayer culture and during emigration from 3D spheroids with respect to cell survival, cell and cytoskeletal orientation, distribution, and expression profile. Monolayers and spheroids were cultured in crosslinked polydimethyl siloxane (PDMS) elastomeric chambers and uniaxially stretched (14% at 0.3 Hz) for 48 h. Cell vitality, their distribution, nuclear shape, stress fiber orientation, focal adhesions, proliferation, expression of ECM components such as sulfated glycosaminoglycans, collagen type I, decorin, tenascin C and cell–cell communication-related gap junctional connexin (CXN) 43, tendon-related markers Mohawk and tenomodulin (myodulin) were analyzed. In contrast to unstretched cells, stretched fibroblasts showed elongation of stress fibers, cell and cytoskeletal alignment perpendicular to strain direction, less rounded cell nuclei, increased numbers of focal adhesions, proliferation, amplified CXN43, and main ECM component expression in both cultures. The applied cyclic stretch protocol evoked an anabolic response and enhanced tendon-related marker expression in ACL-derived fibroblasts emigrating from 3D spheroids and seems also promising to support in future tissue formation in ACL scaffolds seeded in vitro with spheroids.

## Introduction

Tendon and ligaments are mechanosensitive tissues with a very similar histoarchitecture. Both consist mainly of ECM comprising abundant bundles of collagen fibers, oriented parallel to the main direction of strain with only few cells communicating with each other via gap junctions localized at their cell extensions with which cells surround bundles of collagen fibers (Benjamin and Ralphs 1997). In the relaxed ligament, the ECM fascicles form wavy crimps, since they contain also some elastic fibers (Duthon et al. 2006). The majority of ligament-derived cells are fibroblasts which are strictly arranged in longitudinal rows (Hoffmann and Gross 2007). Adequate repetitive mechanical impulses are necessary for tenogenic/ligamentogenic commitment, differentiation of precursor cells and maintenance of the differentiated phenotype (Govoni et al. 2016; Wang et al. 2013) as well as cell survival (Arnoczky et al. 2008; Egerbacher et al. 2008). Especially, the anterior cruciate ligament (ACL) as an intraarticular ligament sustained a constant load up to 1800 N (human ACL) before it ruptures (Rathbone and Cartmell 2011). A stretch of the posterolateral bundle of the ACL can be observed in knee extension while the anteromedial bundle is getting taut in knee flexion (Girgis et al. 1975). Hence, during physiological activities, the ACL-derived fibroblasts are subjected to permanent mechanical stimulation and for in vitro culturing of them the application of physiologically relevant mechanical stimuli is highly recommended to mimic in vivo conditions more closely. Moreover, in several studies cyclic strain was sufficient to enforce in vitro mesenchymal precursor cells into tenogenic lineage differentiation not only in 2D cultures (Chen et al. 2012) but also in 3D scaffolds (Altman et al. 2002; Kuo and Tuan 2008). In response to changed biomechanical conditions, connective tissue fibroblasts are able to adapt collagen synthesis and fiber alignment according to the changed biomechanical conditions (Ishigaki and Kubo 2018; Kessler et al. 2001; Kim et al. 2002; Kjaer et al. 2015). Especially the gene expression of collagen types I and III is increased by uniaxial stretch probably through the autocrine secretion of transforming growth factor (TGF)-β1 (Kim et al. 2002). The regulation of further ligament associated ECM components such as decorin (DCN), tenascin C (TNC), tenomodulin (TNMD) and the tendon/ligament transcription factors, scleraxis (SCX), and early growth response protein (EGR)1 is also responsive to mechanical stimulation (Liu et al. 2017; Nam et al. 2015; Yang et al. 2019; Zhang et al. 2008). Moreover, mechanical stimuli are known to lead to tenocyte proliferation through an autocrine loop triggered by substance P (Backman et al. 2011). Cyclic stretching induces cell and cytoskeleton reorientation, especially of the actin filaments in tendon and skin derived fibroblasts, as well as smooth muscle cells (Dartsch and Hämmerle 1986; Neidlinger‐Wilke et al. 2001; Wang et al. 2005), and induced an upregulation of the specific myofibroblast marker alpha-smooth muscle actin (αSMA) in patellar tendon-derived fibroblasts (Wang et al. 2005). Reorientation behavior is even more pronounced in cell sheets compared to separated cells (Noethel et al. 2018). Furthermore, 2D cell culture can lead to a loss of cellular function, cell response and, thus, also to a reduction of tissue specific gene expression (Ma et al. 2013; Orsini et al. 2018). For this reason, 3D cultures based on spheroids are generated to mimic the native tissue (Friedrich et al. 2009; Tung et al. 2011). Simplicity, reproducibility, and similarity to vital tissues are the main arguments for using 3D cell clusters such as spheroid cultures, which represent one of the most well-characterized 3D ligament fibroblast models (Hoyer et al. 2015; Mueller-Klieser 1997; Schwarz et al. 2019). Spheroid self-assembly is mediated by cell–cell interaction, and resulting micro-tissues are able to produce ligament-specific ECM (Hoyer et al. 2015; Mueller-Klieser 1997; Schwarz et al. 2019). Our experiments using ACL spheroids were performed in Geltrex®-coated crosslinked polydimethylsiloxane (PDMS) chambers. The advantage of PDMS is that it provides a transparent, inert, and non-cytotoxic highly extensible surface of durable elasticity suitable for proliferation of mammalian cells such as osteoblasts (Jeon and Kim 2012), embryonic stem cells (Eroshenko et al. 2013), and neural cell differentiation of umbilical cord blood-derived mesenchymal stem cells (Kim et al. 2008). A higher compared to lower stiffness of PDMS substrates stimulates survival and proliferation of L929 mouse fibroblasts (Park et al. 2010). It has additionally been reported that mechanical strain promotes fibroblast adhesion, growth and increased ECM production on PDMS chambers (Cui et al. 2015; Wang et al. 2015). However, it is still unclear which mechanical stretching parameters like amplitude, frequency, and number of cycles stimulate fibroblasts in a way that they produce a maximum of ECM proteins. Until now, mechanically induced effects have not been investigated in detail in ACL-derived fibroblasts and directly compared between 2D cultures and cells emigrating from 3D spheroid cultures. Therefore, the aim of the present study was to characterize the effect of cyclic uniaxial stretch on ACL-derived fibroblast 2D monolayer culture as well as on cells emigrating from 3D spheroid cultures. Cells emigrating from 3D spheroid cultures were investigated as their transcriptome and proteome might resemble more closely in vivo conditions (Schwarz et al. 2019) than monolayer cultured cells, and they can successfully be used for directed scaffold seeding for ligament tissue engineering (Schwarz et al. 2019). Cells within spheroids are surrounded by their own freshly produced tissue-specific ECM, and the intimate cell-ECM interaction might prime the cells and, thereby, stabilize their tissue-specific expression profile.

Cell survival, morphology, cell and cytoskeletal organization, migration, as well as proliferation were evaluated. Not only the expression of ECM molecules, such as collagen type I, DCN, TNC, and TNMD, but also the cell–cell communication mediating protein CXN43 and transcription factor MKX were determined by gene expression analysis.

## Materials and methods

### 6× cell stretcher

**Fig. 1:**
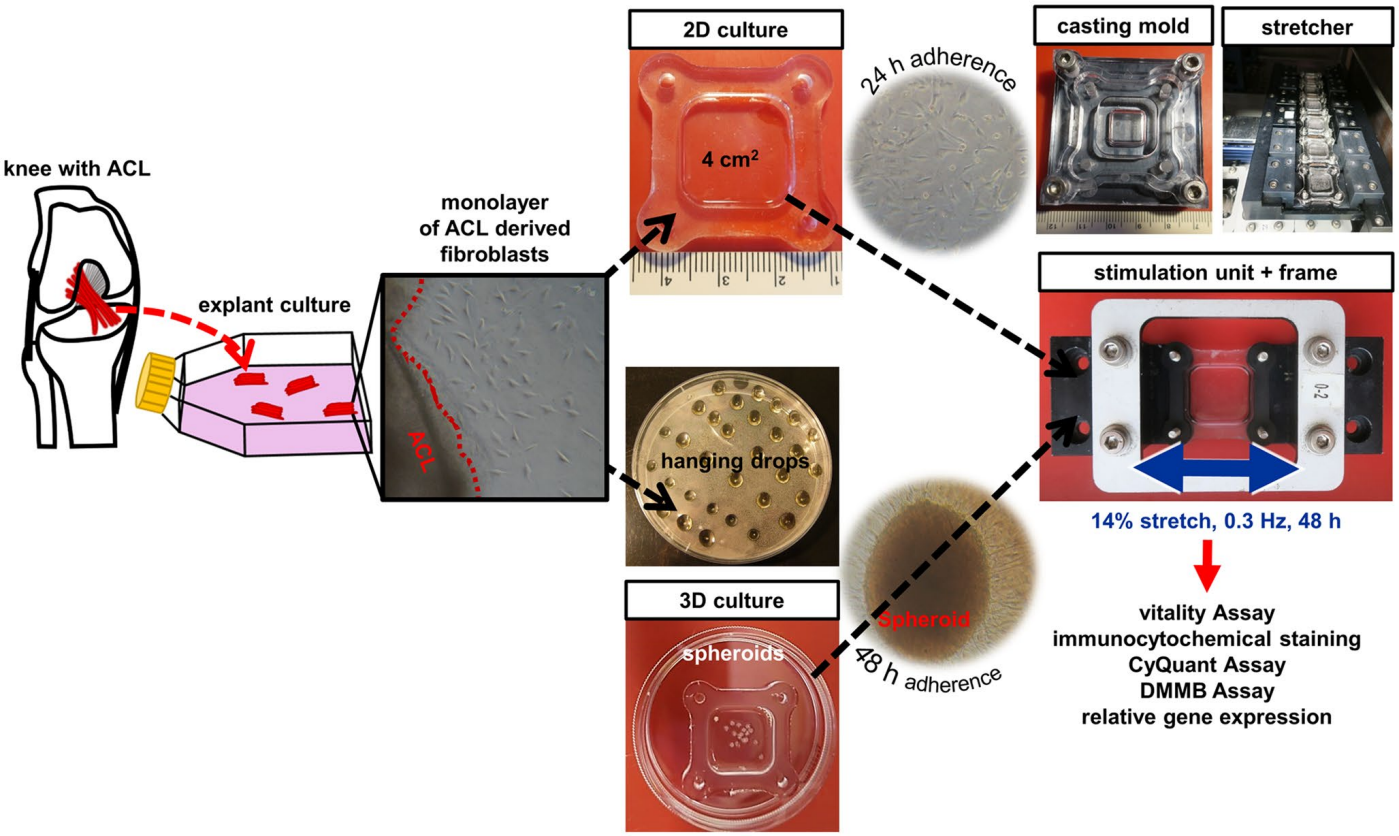
Graphical abstract. After dissection of the anterior cruciate ligament (ACL), 2 mm^2^ tissue pieces were placed in a culture flask as an explant culture. After few days, fibroblasts emigrated out of the ACL. In the first approach, fibroblasts were seeded directly into a 4 cm^2^ PDMS silicone elastomeric chamber as a monolayer (2D) culture and after 24 h of adherence, the stretch program (14% stretch, 0.3 Hz for 48 h) started. In the second approach, spheroids (sph) (3D culture) were assembled using the hanging drop method and after 48 h of adherence they were stretched with the Cell-Stretcher X6 (blue double-headed arrow: direction of stretching). Downstream analyses included vitality Assay, immunocytochemical staining, CyQuant Assay, dimethylmethyleneblue (DMMB) Assay and relative gene expressions of ligament-related components were performed after 2 days of stimulation

For cell stretching experiments a custom-made 6× cell stretcher equipped with a linear stepper motor (MT63, Steinmeyer Mechatronik GmbH, Dresden, Germany) and controlled by LabVIEW software (version 2.0) was used as described previously (Kubo et al. 2020). The whole stimulation system could be placed in the incubator. It contained six elastomeric chambers consisting of crosslinked PDMS for cell cultivation (2 × 2 cm inner width, culture area 4 cm^2^, thickness of the stretched bottom of the chamber 0.5 mm, outer rim 0.5 cm thick and 4 mm high) (Fig. 1). Each chamber was fixed along the complete rim axes perpendicular to stretch direction. Before each experiment, the mechanical stretcher was calibrated at the zero-position using a calibration plate. All components of the 6× cell stretcher were sterilized with isopropanol before assembly under sterile conditions. Non-stretched control cells were cultured in the same chambers under identical conditions.

### Preparation of PDMS chambers

Elastomeric chambers were made of Sylgard 184 (Ellsworth, WI, USA) in a base to crosslinker ratio of 40:1 as described previously (Faust et al. 2011). After transferring into a 50 mL syringe and degassing, 5 mL of the mixture was molded air bubble-free into each polysterol casting frame (Fig. 1). After crosslinking at 60 °C for 16 h, chambers with a Young’s modulus of 50 kPa were stored at room temperature (RT) and protected from ultraviolet light until use. Prior to cell seeding chambers were sterilized with isopropanol within the stretching frame and dried overnight under the bench.

### Cell isolation and monolayer culture

Lapine ACL-derived ligament fibroblasts were isolated from the ACLs of five healthy, female New Zealand Rabbits (mean age of 12 months) derived from the abattoir. The ACLs were sliced into 2 mm^2^ pieces and placed into a T25 culture flask with growth medium (Dulbecco’s modified Eagle’s medium (DMEM)/Ham’s F12 medium (1:1) (Biochrom AG, Berlin, Germany), supplemented with 10% fetal bovine serum (FBS, Biochrom AG), 1% penicillin/streptomycin solution, 25 μg/mL ascorbic acid (Sigma-Aldrich, Munich, Germany), 2.5 μg/mL amphotericin B (Biochrom AG), 0.5% MEM amino acid solution (100×) (Sigma-Aldrich). Growth medium changes were done every second day. When reaching 80–90% confluence, outgrowing ACL-derived fibroblasts were detached with trypsin/ethylenediaminetetraacetic acid (EDTA) solution (0.05% trypsin/0.02% EDTA (w/v) (Biochrom AG). Cell number as well as viability were determined by trypan blue exclusion stain using a hemocytometer.

### Cell seeding and spheroid fabrication

For the spheroid cultures chambers were coated with Geltrex® (lactose-dehydrogenase elevating virus-free reduced growth factor basement membrane matrix, Gibco, Thermo Fisher Scientific, Darmstadt, Germany). Geltrex® coating was performed for 1 h at RT by using the thin gel method according to the manufacturer’s instructions. For this purpose, thawed Geltrex® solution was slowly pipetted up and down and subsequently mixed with Hank’s Balanced Salt Solution with Ca^2+^/Mg^2+^ (HBSS, Carl Roth, Karlsruhe, Germany) for a final concentration of 9 mg/mL. The chambers were incubated for 1 h at RT with 50 µL/cm^2^ of Geltrex® solution. For monolayer formation, 6000 ACL-derived fibroblasts/cm^2^ were seeded into uncoated PDMS chambers (without Geltrex®) with 1000 µL growth medium containing 10% FBS. After 24 h of adherence, the PDMS chambers with fibroblasts were stretched for 48 h with an amplitude of 14% and a frequency of 0.3 Hz, a speed of 12% of the chamber length per second (2.4 mm/s) and a dwell time of 0.417 s. Spheroids consisting of 2.5 × 10^4^ cells were produced by using the hanging drop method. Therefore, the cells were trypsinized, washed with PBS and resuspended in 10% FBS containing growth medium to a final concentration of 2.5 × 10^4^ cells per 50 µL drop. Cell suspension was dropped as single drops onto the inner surface of the lids of petri dishes with 90 mm diameter (nerbe plus GmbH, Winsen, Germany), which were inverted over the bottom of the dishes that contained PBS to prevent drying. After 48 h of spheroid formation in the incubator at 37 °C and 5% CO_2_, the spheroids were harvested and placed into a Geltrex® coated PDMS chamber with a maximum of 100 µL growth medium. After 24 h of adherence 900 µL growth medium were added into the chambers and after additional 24 h of incubation, the PDMS chambers were stretched with the same stimuli as the monolayer cultures.

### Cell vitality analysis

To examine the vitality of the cells after cultivation (unstimulated vs. stimulated), live/dead staining using 1 µL propidium iodide (PI, 1% stock solution) (Thermo Fisher Scientific), and 5 µL fluorescein diacetate (FDA, stock solution: 3 mg/mL in acetone, Sigma-Aldrich) in 1 mL 1× phosphate- buffered saline (PBS) was performed. For implementation, growth medium was removed from the seeded PDMS chambers and 50 µL of stain solution were applied. After a 5-min incubation period at RT, the fluorescence of live and dead cells was monitored using a Leica TC SPEII confocal laser scanning microscope (CLSM, Leica, Wetzlar, Germany). Diameters of FDA stained spheroid were measured using the CLSM. Based on the results of the live/dead assay, the area covered by vital cells was calculated with ImageJ1.48 v software (U.S. National Institutes of Health, Bethesda, MD, USA). Three independent experiments with cells derived from three different donors were performed.

### Quantitative measurements of DNA and sGAG content

By CyQUANT® NF Cell Proliferation Assay, the influence of respective stimulation on cell proliferation was examined after the cultivation time (72 h for 2D and 96 h for 3D culture). After cultivation, growth medium was completely removed, and cells (either monolayers or spheroids together with emigrating cells) were washed carefully with HBSS. Subsequently, HBSS was discarded and 50 µL of the dye solution (HBSS + dye binding solution 1:500) was applied to each seeded well. The standard curve was generated by serial dilution of calf thymus DNA stock solution (1 mg/mL) with TRIS/EDTA (TE) buffer (TRIS EDTA buffer: 10 mM TRIS [pH 8.0] and 1 mM EDTA in H_2_O_deion__._). For the standard curve, 25 µL of the serial calf thymus DNA dilutions was mixed with 25 µL of CyQuant dye solution (HBSS + dye binding solution 1:250, Thermo Fisher Scientific). Subsequently, plates were covered to be protected from light and incubated at 37 °C for 60 min. The fluorescence of each well was measured in triplicates at 485 nm excitation/530 nm emission in a fluorometric plate reader (Tecan, Groedig, Austria). For the dimethyl methylene blue (DMMB) assay, the same supernatant was used as in the CyQuant assay. After adequate sample dilution, the DMMB (AppliChem, Darmstadt, Germany) was added consisting of 40 mM glycine (Sigma-Aldrich), 40 mM NaCl (Carl Roth GmbH) at pH 3 and DMMB (8.9 mM in ethanol). Chondroitin sulfate (Sigma-Aldrich) was used as standard. The absorption shift was measured at a wave length of λ = 633 nm to λ = 552 nm using a genius spectral photometer (Tecan). Three independent experiments with cells derived from three different donors were performed.

### Immuncytochemical staining

The protein expression profile was assessed using CLSM. Monolayer and spheroid cultures (*n* = 3) were fixed in 4% paraformaldehyde (PFA), washed with Tris-buffered saline (TBS: 0.05 Tris, 140 mM NaCl, pH 7.6), before incubation with blocking buffer (5% protease free donkey serum diluted in TBS with 0.1% Triton X-100 for cell permeabilization) was performed for 20 min at RT. Primary antibodies used were initially tested for specificity by direct comparison of immunoreactivity in human- and rabbit-derived ACL-derived fibroblasts (data not shown). Samples were incubated with primary antibodies (see Table 1: mouse-anti-human αSMA, goat-anti-human collagen type I, mouse-anti-human ki67 and mouse-anti-human paxillin) for one hour at RT. After rinsing with TBS samples were incubated for 1 h with donkey-anti-goat (Invitrogen, Carlsbad, USA) or donkey-anti-mouse-cyanine-3-(Cy3, Invitrogen) coupled secondary antibodies (diluted 1:200 in blocking buffer see Table 1) at RT. The cell nuclei were counterstained using 10 µg/mL 4′,6′-diamidino-2-phenylindol (DAPI, Roche, Mannheim, Germany).

**Table 1:** Antibodies used for immunocytochemistry

Target	Primary antibody	Dilution	Secondary antibody	Dilution
Ki67	mouse-anti-human, Millipore (MAB4190)	1:30	donkey-anti-mouse Cy3, Invitrogen	1:200
Paxillin	mouse-anti-human, BD Biosciences (610052)	1:40	donkey-anti-mouse Cy3, Invitrogen	1:200
Collagen type I*	Goat-anti human, Biozol (1310–01)	1:30	donkey-anti-goat Alexa Fluor 488, Invitrogen	1:200
αSMA*	mouse-anti-human, Sigma-Aldrich (A5228)	1:50	donkey-anti-mouse Cy3, Invitrogen	1:200

^*^Immunoreactivity in rabbit-derived cells has already been shown by others (Jerdan, et al. 1991; Sharawy, et al. 2003; van Royen, et al. 2002)

Phalloidin-Alexa-Fluor 488 (1:100, Santa Cruz Biotechnologies, Inc., TX, USA) was used to depict the F-actin cytoskeletal architecture. After three times of washing with TBS, the immunolabeled cells were mounted with a fluoromount mounting medium (Southern Biothech, Biozol Diagnostica, Eching, Germany) and examined by CLSM.

The length of the F-actin stress fibers was measured with the “straight line” tool from the ImageJ using the F-actin staining as mask. Ki67 positive cells were shown in red and could be distinguished from the “non-dividing” cells, which had cell nuclei only stained in blue due to DAPI. The amount of ki67 positive cells was counted.

The number of focal adhesion sites immunolabeled by paxillin-antibody was calculated. Based on DAPI staining amount, roundness and perimeter of the cell nuclei was measured with ImageJ. Roundness is defined as 4*area/(π*major_axis^2^): a perfect circle has a roundness of 1. A roundness index close to 1 indicates that a nucleus has few extensions, whereas an index value closer to 0 corresponds to an ellipsoid nuclear shape. For collagen type I and αSMA, fluorescence intensities were measured using ImageJ.

Three microscopic fields of each independent experiment (*n* = 3, with cells from three different donors) were included in all calculations.

### RNA isolation

Cells colonizing the PDMS chambers (unstimulated and stimulated, monolayer and spheroid cultures) were lysed in RLT buffer (Qiagen, Hilden, Germany) + 1% mercaptoethanol (Carl Roth) and detached with a cell scraper. RNA was isolated and purified using the RNeasy Mini kit according to the manufacturer´s instructions (Qiagen), including on-column DNAse treatment. Quantity and purity of the RNA samples were monitored (260/280 absorbance ratio) using the Nanodrop ND-1000 spectrophotometer (Peqlab, Biotechnologie GmbH, Erlangen, Germany).

### Quantitative real-time PCR

For cDNA synthesis 120 ng of total RNA were reverse transcribed using the QuantiTect Reverse Transcription Kit (Qiagen AG) according to supplier´s manual. Twenty nanograms of cDNA were used for each quantitative real-time PCR (qRT-PCR) reaction as triplicate using TaqMan Gene Expression Assays (Life Technologies) with primer pairs for type I collagen (*COL1A1*, Oc03396073_g1), decorin (*DCN*, Hs00370384_m1), tenascin C (*TNC*, Oc06726696_m1), connexin 43 (*CXN43*, Oc03396056_g1), Mohawk (*MKX*, Oc06754037_m1), tenomodulin (*TNMD*, Oc03399505_m1 [synonymous: myodulin]), and glyceraldehyde-3-phosphate dehydrogenase [GAPDH (Oc03823402_g1)] was used as a reference gene (Table 2). qRT-PCR was performed using the real-time PCR detector StepOnePlus (Applied Bioscience [ABI], Foster City, USA) thermocycler with the StepOnePlus software 2.3 (ABI). The relative gene expression of the gene of interest was normalized to the GAPDH expression and calculated for each sample using the ∆∆CT method as described previously (Schefe et al. 2006). Five independent experiments with cells derived from five different donors were performed.

**Table 2:** Primers used for gene expression analysis

Gene symbol	Species	Gene name	Amplicon length (base pairs)	Assay ID from ABI
COL1A1	*O. cuniculus*	Collagen type I alpha 1	70	Oc03396073_g1
DCN	*Homo sapiens*	Decorin	77	Hs00370384_m1
TNC	*O. cuniculus*	Tenascin C	61	Oc06726696_m1
CXN43	*O. cuniculus*	Connexin 43	68	Oc03396056_g1
MKX	*O. cuniculus*	Mohawk	60	Oc06754037_m1
TNMD (LOC100125994)	*O. cuniculus*	Tenomodulin	146	Oc03399505_m1
GAPDH	*O. cuniculus*	Glyceraldehyde-3- phosphate dehydrogenase	82	Oc03823402_g1

*O Oryctolagus*

### Statistics

Amount, roundness, and perimeter of the cell nuclei were statistically evaluated with IBM®SPSS Statistics Version 24.0.0.32-Bit Version. Values were expressed as mean with standard deviation using GraphPad Prism 8 (GraphPad Software Inc., San Diego, CA, USA). Data were tested for normal distribution using the Kolomogorov Smirnov test (α = 0.05). For analysis of normal distributed data, the unpaired two-sided *t* test and the one-way ANOVA with subsequent Holm-Sidak adjustment were used. Statistical significance was set at a *p* value ≤ 0.05 (*), *p* value ≤ 0.01 (**), *p* value ≤ 0.001 (***), and *p* value ≤ 0.0001 (****). Three or five independent experiments with cells derived from three or five different donors were performed.

## Results

### Cyclic stretching induced morphological and cytoskeletal changes

#### Cell morphology and F-actin cytoskeleton

**Fig. 2:**
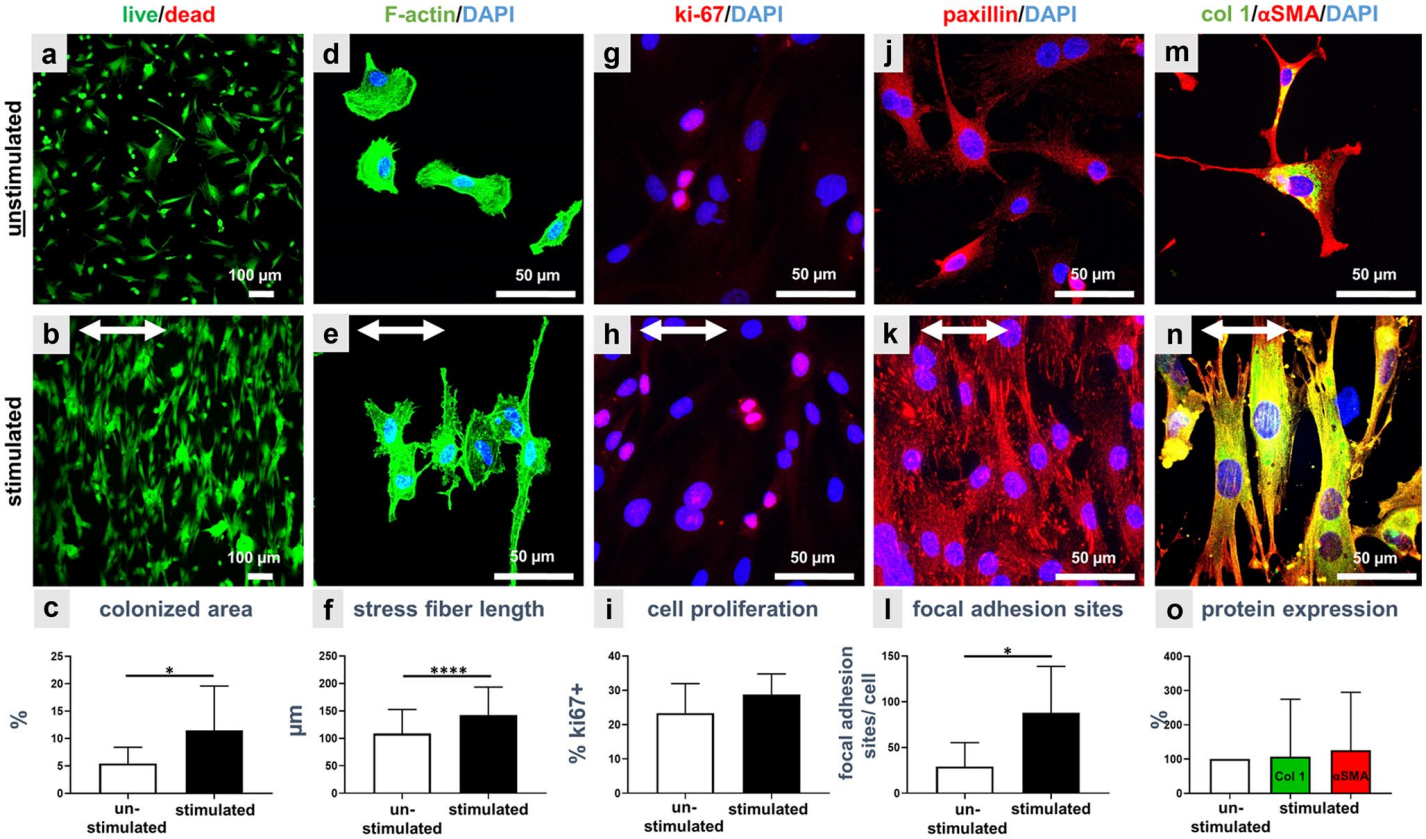
Monolayer culture of unstimulated (**a**, **d**, **g**, **j**, **m**) and stimulated (**b**, **e**, **h**, **k**, **n**) ACL-derived fibroblasts after 48 h of 14% stretch. The vitality assay (**a**, **b**) showed live (green) and dead (red) cells and based on living cells the colonized area could be calculated (**c**). The phalloidin Alexa 488/ DAPI staining (**d**, **e**) depicted the F-actin (green) and cell nuclei (blue); the elongation of the stress fibers was measured (**f**). The immunocytochemical staining showed the proliferation marker ki67 (**g**, **h**, red) and the amount of ki67+ cells was calculated (**i**). Paxillin (**j,** **k**) is depicted in red, collagen type I (**m, n**) in green and alpha smooth muscle actin (αSMA [**m, n**]) in red. The cell nuclei were stained with DAPI in blue. Mean number of focal adhesion sites per cell (**l**) and the relative protein expression of collagen type I (col 1) and αSMA (**o**) were determined versus controls which were normalized. Three independent experiments were performed with significances (*) of *p* ≤ 0.05 and (****) of *p* ≤ 0.0001. The stretch direction is indicated with the double-headed arrows (**b**, **e**,** h**, **k**, **n**). Scale bars 100 µm (**a**, **b**), 50 µm (**d**, **e**, **g**, **h**,** j**, **k**, **m**, **n**)

The vitality assay showed that the majority of ACL-derived fibroblasts adhered and survived on the PDMS surface not only in the unstimulated but also in the stimulated PDMS chambers (Fig. 2a, b) after 14% stretch for 48 h. Cell clustering, flattened and elongated cell shapes could be observed in the stimulated cultures. Unstimulated cells had polymorphic fibroblastic shapes and were evenly and randomly distributed in the PDMS chambers (Fig. 2a). Neither unstimulated nor stimulated ACL-derived fibroblasts utilized in this study had lost their fibroblast-like morphology. Stretched fibroblasts preferentially orientated in perpendicular direction relative to stretch and had a spindle-shaped morphology (Fig. 2b). The calculation of the colonized area was done for the vital cells (Fig. 2c) and showed a significantly smaller surface covered with fibroblasts in unstimulated (5.2% colonized area) compared to stimulated (11 ± 5% colonized area) cultures. A more detailed view on cell shape and size was shown by F-actin staining (Fig. 2d, e). Unstimulated cells (Fig. 2d) were larger and had a more spread cell shape in comparison to the stimulated ones. The stimulated ACL-derived fibroblasts had an elongated and slimmer cell body, which was orientated with most of its cytoskeletal F-actin fibers perpendicular to the strain direction (Fig. 2e). Stress fibers in unstimulated cells were in the mean about 91 ± 33 µm long and significantly shorter than stress fibers in stimulated cells (150 ± 37 µm) (Fig. 2f).

#### Strain enhances cell adhesion

**Fig. 3:**
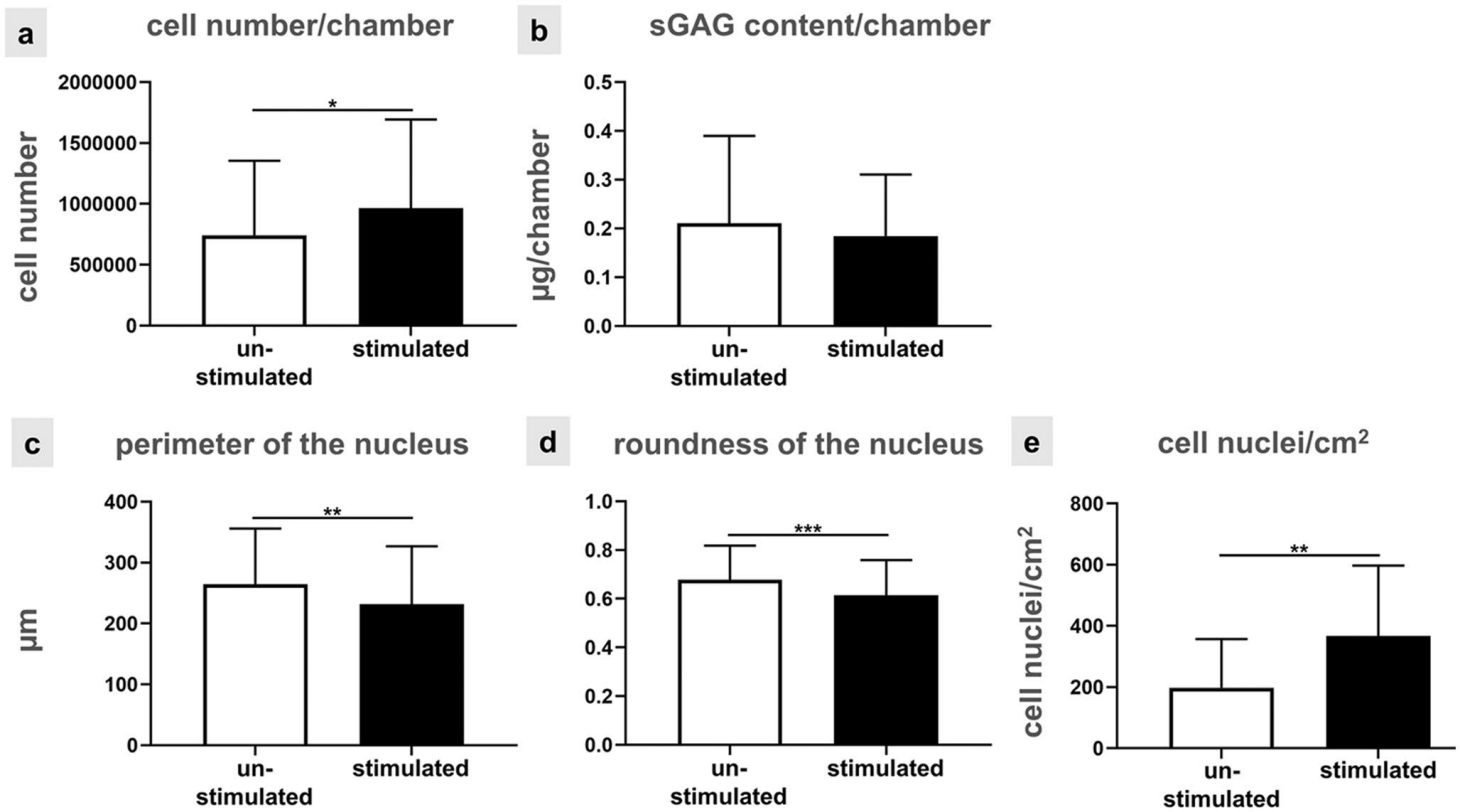
Evaluation of the cell response to mechanical stretch in the monolayer culture. Cell proliferation assay (**a**), a calculation of the sGAG content (**b**), and the measurement of nuclear shape, especially the mean values of the perimeter of the nucleus (**c**) and mean values of the roundness of the nuclei (**d**) were measured and calculation of number of cell nuclei using DAPI staining (**e**). Three independent experiments were performed with significances (*) of p ≤ 0.05, (**) of *p* ≤ 0.01 and (***) of *p* ≤ 0.001

Immunolabeling of the proliferation marker ki67 (Fig. 2g, h) showed that stimulated cells tended to proliferate more (28 ± 4% ki67-positive cell nuclei in mean) than unstimulated fibroblasts (23 ± 7%) (not significant, Fig. 2i). Focal adhesion sites mediating cell adhesion to PDMS were depicted by the paxillin immunolabeling. While focal adhesion sites were weakly detectable in unstimulated ACL-derived fibroblasts, stimulated fibroblasts developed clearly visible adhesions (Fig. 2j, k). Quantitative analysis resulted in significantly higher numbers (88 ± 48) of focal adhesion sites per cell in stimulated cells compared to unstimulated fibroblasts, where only 29 ± 24 focal adhesion sites were counted (Fig. 2l). The main ligament ECM component collagen type I could be demonstrated not only in the unstimulated but also in the stimulated PDMS chambers (Fig. 2m, n). The immunolabeling of collagen type I did not significantly differ between unstimulated and stimulated ACL derived fibroblasts (Fig. 2m, n). Collagen type I immunoreactivity was mainly intracellularly detected, especially in the perinuclear rough endoplasmatic reticulum region of the cells (Fig. 2n). The typical myofibroblast marker αSMA was expressed in both, unstimulated and stimulated fibroblasts (Fig. 2m, n). The calculation of the relative collagen type I and αSMA protein expression in controls vs. stimulated cells revealed no significant difference (Fig. 2o). Significant differences in cell number per chamber could be evaluated with the CyQuant Assay based on DNA content (Fig. 3a) but also based on DAPI staining (Fig. 3e). A stimulation of fibroblasts led to a significantly higher DNA content and significantly higher number of cell nuclei (Fig. 3a, e) in comparison to unstimulated ones. The sulfated glycosaminoglycan (sGAG) content per chamber was not significantly different between unstimulated and stimulated cells (Fig. 3b). Morphological changes in size/shape of the cell nuclei in response to mechanostimulation were calculated with ImageJ. Results showed that cell nuclei of unstimulated cells had a significantly larger perimeter (335 ± 79 µm) in comparison to stimulated fibroblasts (202 ± 45 µm) (Fig. 3c). Mechanostimulation induced not only a change in perimeter but also in roundness of cell nuclei: unstimulated fibroblast cell nuclei were significantly rounder (0.69 ± 0.10) than stimulated ones (0.66 ± 0.11) (Fig. 3d).

### Strain enhances ligament-related gene expression in monolayer cultured cells

**Fig. 4:**
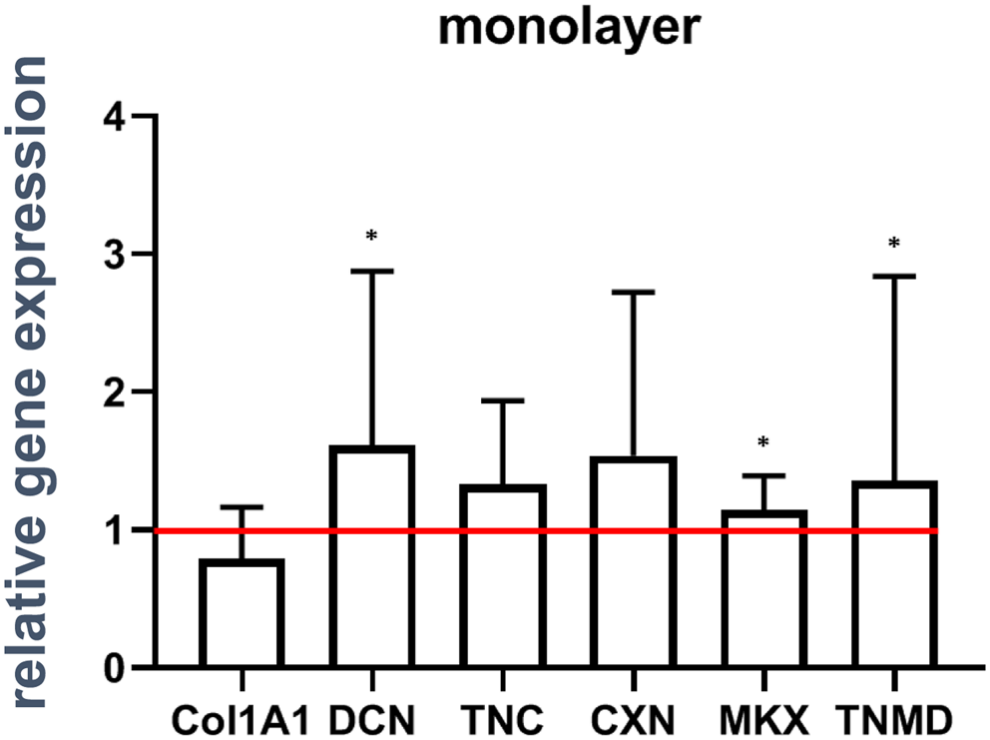
Expression levels of genes coding for ligament-related components in the monolayer culture. Relative gene expression of collagen type I (COL1A1), decorin (DCN), tenascin C (TNC), connexin 43 (CNX), Mohawk (MKX), and tenomodulin (TNMD) were shown with standard deviation. The red line depicts the normalized level of unstimulated controls. Five independent experiments were performed with significances (*) of *p* ≤ 0.05

To determine if uniaxial cyclic stretch influences the gene expression of ligament-related components in ACL-derived fibroblasts, PCR analyses were performed (Fig. 4). Both, unstimulated and stimulated ACL-derived fibroblasts expressed mRNA of typical ligament ECM components like COL1A1, DCN, and TNC. Differences in the gene expression of COL1A1, TNC, gap junction component CXN43 did not reach the significance level in unstimulated compared to stimulated fibroblasts. However, the proteoglycan DCN, MKX and the ligament-related glycoprotein TNMD were significantly higher expressed after mechanostimulation in monolayer culture compared to unstimulated controls.

### Cell emigrating from spheroids during stretching

#### Spheroid shape and ECM composition

**Fig. 5:**
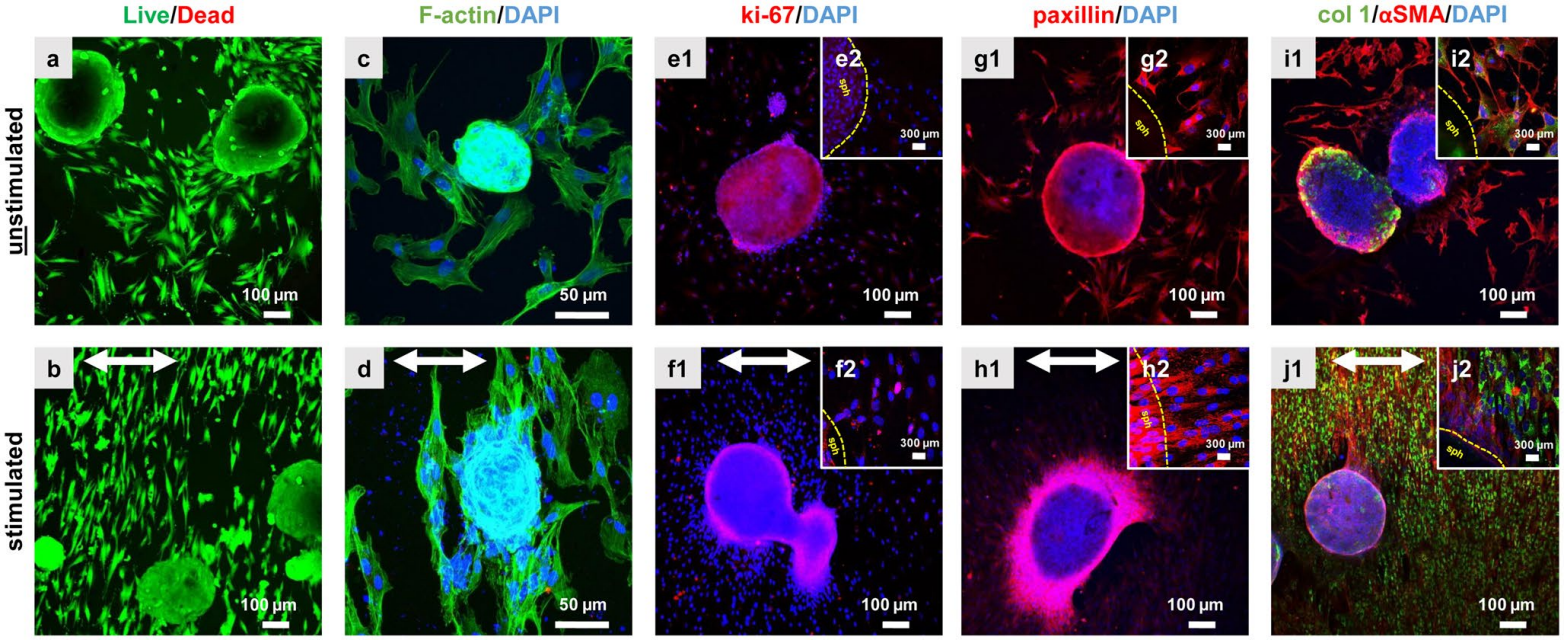
Unstimulated (**a, c, e, g, i**) and stimulated (**b, d, f, h, j**) ACL-derived fibroblasts emigrating from spheroids after 48 h and 14% stretch at 0.3 Hz. The vitality assay (**a, b**) showed live (green) and dead (red) cells. The phalloidin Alexa 488/ DAPI staining (**c, d**) depicted the F-actin (green) and cell nuclei (blue). The immunocytochemical staining visualized the proliferation marker ki67 (**e, f**) in red, paxillin (**g, h**) in red, collagen type I **(i, j**) in green and αSMA (**i, j**) in red. Small inserts showed a higher magnification (**e2, f2, g2, h2, i2, j2**) of the cells at the border of the spheroids (sph). The cell nuclei were stained with DAPI in blue. Scale bars 100 µm (**a, b**), 50 µm (**c–j**). The stretch direction is indicated with the double-headed arrows (**b, d, f, h, j**)

**Fig. 6:**
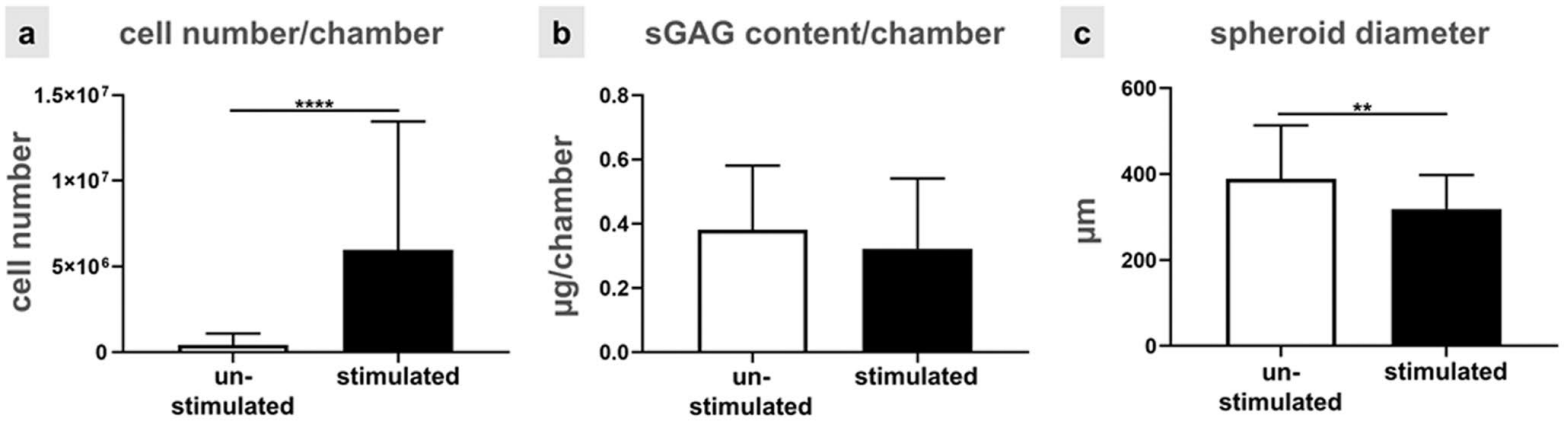
Evaluation of the cell response to mechanical stretch in spheroid-derived fibroblasts. Mean values of the cell proliferation assay (**a**) and of the sGAG content (**b**) were shown. Additionally, the spheroid diameters were determined (**c**). Three independent experiments were performed with significances (**) p ≤ 0.01 and (****) of *p* ≤ 0.0001

Cells emigrating from spheroids reorganize their cytoskeleton, proliferation, and adapt their expression profile in response to strain. Ligaments are inherently 3D tissues, so the question was addressed whether ACL derived fibroblasts might respond differentially to the same stimulation protocol depending on being either already maturated in 3D environments before emigration from 3D spheroids and stimulation or being exposed as monolayer to similar conditions. For a better comparison between both conditions the same evaluations (vitality assay, F-actin staining, ki67, paxillin, collagen type I, and αSMA immunolabelings) were performed. The majority of the spheroid-derived fibroblasts was still alive irrespectively whether being stretched for 48 h or not. Not only the vitality assay but also the immunostaining showed the cells migrating out of the spheroids under both conditions (Fig. 5). A random migration out of the spheroids could be observed in the unstimulated fibroblasts (Fig. 5a), while a synchronized cell orientation perpendicular relative to the direction of stretch could be seen in the stimulated chambers (Fig. 5b). Detection of stress fibers in the cells emigrating from the spheroids showed, similar to monolayer conditions, more elongated cell shapes and a cell orientation perpendicular to strain direction in stimulated compared to unstimulated cells (Fig. 5c). Furthermore, it seemed that straining of ACL derived fibroblasts led to an increase in numbers of emigrating cells (Fig. 5b, d). Based on the ki67 staining, there was a higher proliferation rate detectable in the center of the unstimulated spheroid cell clusters (Fig. 5e1) and no proliferation at the border of the spheroid as indicated by higher magnification (Fig. 5e2). In contrast, stimulated spheroids revealed cells proliferating at their margins (Fig. 5f1) and emigrating fibroblasts proliferating near to the spheroid border (Fig. 5f2). Paxillin-positive focal adhesion sites were detectable only at the margins of unstimulated spheroids (Fig. 5g1), and also just weakly expressed in a few emigrating cells (Fig. 5g2). Vice versa, stimulated spheroid cultures showed that spheroid derived cells exhibited more focal adhesion sites not only at the border of the spheroid but also at emigrating cells (Fig. 5h1). This could be seen more clearly at higher magnification (Fig. 5h2). Additionally, collagen type I was expressed at higher levels only at the border of the unstimulated spheroids than in the few emigrating fibroblasts (Fig. 5i). Hence, the collagen type I production was not homogenously distributed. The stimulated spheroids (Fig. 5j) produced less collagen type I in the spheroid while surrounding cells expressed clearly more intracellular collagen than the unstimulated cells. αSMA expression could be observed not only in the unstimulated but also in stimulated spheroids and their emigrating fibroblasts (Fig. 5i, j). The DNA content of the spheroid colonized chambers including the emigrating cells, was measured after 48 h of uniaxial cyclic stretch. Significant differences in cell number per chamber were detected between unstimulated and stimulated ACL-derived fibroblasts suggesting enhanced cell proliferation in response to strain (Fig. 6a). The sGAG content did not significantly differ between unstimulated (0.35 ± 0.18 µg/chamber) and stimulated spheroids (0.28 ± 0.12 µg/chamber) (Fig. 6b). However, the size of the spheroids decreased significantly after 48 h of cyclic strain (Fig. 6c).

#### Strain elevates ligament-related gene expression in spheroid-derived fibroblasts

**Fig. 7:**
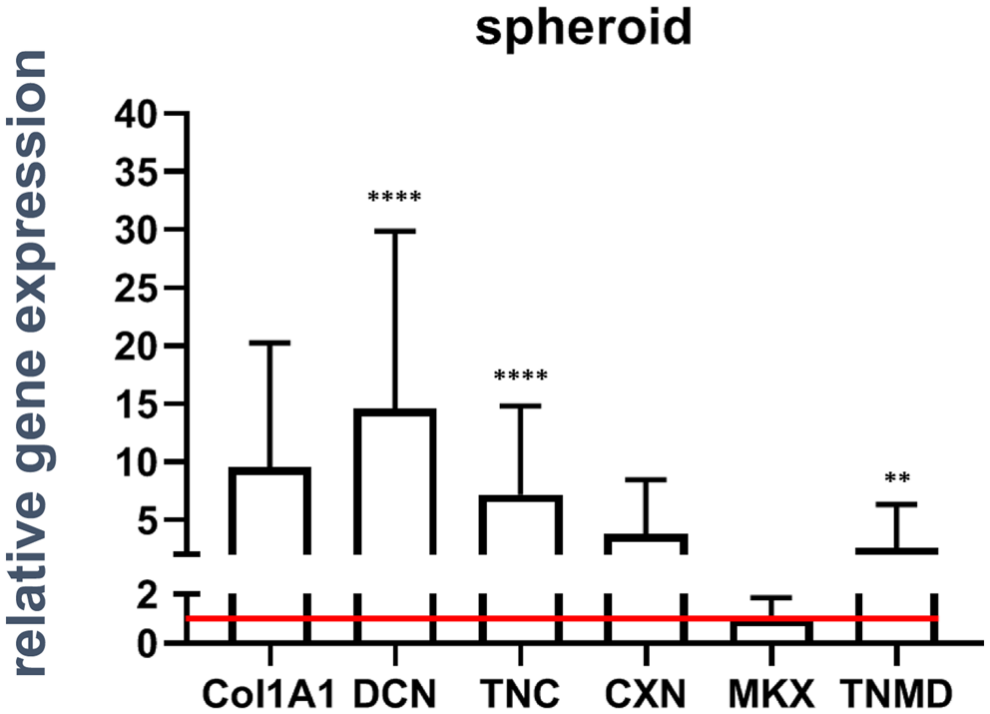
Expression level of genes coding for ligament-related components in spheroid-derived fibroblasts. Relative gene expression of collagen type I (COL1A1), decorin DCN, tenascin C (TNC), connexin (CXN) 43, Mohawk (MKX), and tenomodulin (TNMD) were shown with standard deviation. The red line depicts the normalized level of unstimulated controls. Five independent experiments were performed with significances (**) of p ≤ 0.001 and (****) of *p* ≤ 0.0001

To examine the effect of mechanical stimulation on ACL spheroids, the relative gene expression of ligament-associated genes was detected in unstimulated and stimulated spheroid cultures (Fig. 7). The relative gene expression of COL1A1, DCN, TNC, CXN43, MKX and TNMD was elevated after cyclic stimulation. This increase was not significant for COL1A1 and CXN43 but significant for DCN, TNC, and TNMD. The relative gene expression of MKX showed no changes between unstimulated and stimulated fibroblasts.

## Discussion

The ACL has a strong and compact ECM. It connects the femur with the tibia and is, therefore, one of the most important ligaments for stabilizing the knee joint. Mechanical stretch is a key regulator for cell morphogenesis and contributes to homeostasis of healthy tissues. There exist cell source specific differences in the mechanoresponse as reported for medial collateral ligament- and ACL-derived fibroblasts as well as MSCs (Hsieh et al. 2000; Sun et al. 2016). In this study, ACL-derived fibroblasts were exposed in vitro to a uniaxial stretch. The rate of stretching (14%, 0.3 Hz) was within the range tested as stimulatory signal and not detrimental with ACL fibroblasts in another study (Sun et al. 2016). In the study presented here, ACL-derived fibroblast behavior after maturation in both 2D and 3D culture systems were directly compared under comparable cyclic uniaxial stretch conditions to estimate the effect on 2D and 3D preculturing on the specific mechanoresponse. Since various cell types, including tendon-derived fibroblasts are known to dedifferentiate under 2D conditions and change their functionality (Stoll et al. 2010; Yao et al. 2006), in the last few years, spheroid and pellet cultures of tendon-derived fibroblasts have been used for the investigation of cell performance under in vitro 3D conditions (de Wreede and Ralphs 2009; Kraus et al. 2017). However, the behavior of cells directly emigrating on substrates with nature-mimicking elasticities from self-assembled ACL cell spheroids as used in this study, subjected in parallel to a further natural cyclic stretching of 14% has not been reported so far. Since ACL-derived fibroblast spheroids can be used for scaffold seeding providing a future approach for establishing tissue engineered ACL implants as reported previously (Hahn et al. 2019; Schulze-Tanzil et al. 2020; Schwarz et al. 2019) their mechanoresponse is of high importance. The influence of mechanical strain on ACL-derived fibroblasts in vitro has been described using a variety of stimulation programs and different stimulation devices (Hsieh et al. 2000; Kim et al. 2002; Miyaki et al. 2001). In addition, surface coatings of the material to which the cells are attached to during stretching differ and accordingly, their influence on cell behavior via stimulation of different integrin subtypes (Riehl et al. 2012). The device used in the present study based on elastomeric silicone chambers has already been intensively characterized in regard to several suitable protocols for stimulation of fibroblasts by other research groups (Faust et al. 2011; Kubo et al. 2020; Niediek et al. 2012) but also endothelial cells (Springer et al. 2019; Zielinski et al. 2018). Applying the silicone chamber for attachment of 3D spheroids requires a surface coating and a longer time of spheroid adherence compared to monolayer culture due to the initially small contact area of the spheroids with the chamber surface. To avoid additives such as basal lamina components of Geltrex® which might influence cell response to stretch by providing chemical signals, the PDMS chambers were only coated, when 3D spheroid cultures were used since fibroblast monolayers revealed sufficient cell adherence and survival on uncoated PDMS. Hence, chambers were coated with Geltrex® to guarantee a quick adherence of the spheroids since the pure silicone does not provide sufficient binding motifs. Geltrex® did not affect the differentiation capability of pluripotent stem cells in other studies (Gandhi et al. 2019). Not only a quick adherence is guaranteed but also the maintenance of the ligament-specific characteristics, such as expression of collagen type I, DCN, TNC, MKX, and TNMD. Another study could also confirm the suitability of Geltrex® as a rapid and reproducible adherence substrate not only for primary epithelial cancer cells in 2D but also for 3D neuronal microisland cultures consisting of neurons surrounded by glia cells (Janik et al. 2016; Ricoult et al. 2012).

ACL-derived fibroblasts respond to uniaxial strain, especially with changes in cell and cytoskeleton orientation (Rathbone and Cartmell 2011). This dynamic process of actin reorganization, such as disassembly and reassembly of stress fibers and focal adhesions is still not fully understood. But cell migration by reorganization of the actin cytoskeleton is mediated by the Rho small G protein family (Abiko et al. 2015; Imamura et al. 1998). The results of the present study, cells aligning themselves and F-actin fibers perpendicular relative to direction of stretch in 2D as well as 3D culture, confirmed the computer-aided prediction of (Ristori et al. 2018) and are in accordance with other theoretical predictions and experimental data of fibroblast 2D culture using the same stretching device (Faust et al. 2011). Hence, one can assume that stimulated cells avoid direct exposure to stretch (Chatterjee and Gundiah 2019). However, stretching (2.5%, 2 h a day) under 3D conditions led to an alignment of tendon fibroblasts embedded within a collagen gel in the direction of stretch in the study of (Henshaw et al. 2006). This is highly likely because cells were embedded in a uniaxially constrained tissue as described by (Henshaw et al. 2006). The stretched 3D matrix surrounding the cells might enforce the cells to align parallel in rows. When cells are embedded in a biaxially constrained tissue, they do display strain-avoidance in 3D upon uniaxial cyclic stretching (Foolen et al. 2012). Cell alignment in strain direction is a typical feature of native tendons and ligaments. One could assume that the particular situation of tendon fibroblasts surrounded by their natural tendon ECM or by biomaterials mimicking their ECM (such as collagen hydrogels) in vitro could protect them from the direct impact of stretching. In this case, the ECM probably sustains the main load of uniaxial strain. However, this could not be observed in the biomaterial-free 3D spheroid-based approach in the present study looking at the emigrating cells. However, cell arrangement within the spheroids was not studied in detail. The alignment of emigrating cells went along with the alignment of the F-actin cytoskeleton. Not only ligament derived fibroblasts but also human umbilical vein endothelial cells oriented their cytoskeletal stress fibers in perpendicular direction in 2D culture in a time dependent manner (Abiko et al. 2015; Zielinski et al. 2018). These actin filaments are essential for the cellular reorientation under cyclic stretching (Springer et al. 2019). Furthermore, using spheroid cultures the stress fibers of emigrating cells were also orientated in perpendicular direction relative to stretch. In line with other studies on using fibroblasts (Chatterjee and Gundiah 2019), we also detected a significant elongation of the stress fibers of stimulated ACL fibroblasts in 2D monolayer culture.

The effect of mechanical stress on the cell nucleus in 2D monolayer culture showed that its shape was slightly elongated parallel to the cytoskeletal fibers (Ahmed et al. 2010) or, as in the experiments of this study, become smaller and less round in comparison to unstimulated ACL-derived fibroblasts. In response to stretch cells might also exhibit a more convex cell morphology which suggests also less flattened and hence, apparently smaller cell nuclei. We hypothesized that this phenomenon is due to a chromatin condensation supported by the observations of Nagayama and Fukuei in 3T3 mouse fibroblasts (Nagayama and Fukuei 2019). The more rapid proliferation of cells seen here could also lead to smaller sizes of cell nuclei since cells need time to increase their nuclei diameter after mitosis (Webster et al. 2009).

Cell proliferation in 2D as well as in 3D spheroid culture was not only evaluated based on DNA amount, by applying CyQuant Assay, but also by immunocytochemical staining for ki67. While the results of ki67 staining indicated only a trend of increasing proliferation of ACL, the CyQuant Assay and calculation of the number of DAPI stained cell nuclei show a significant increase in cell number and DNA suggesting proliferation. Proliferation of ACL derived fibroblasts tended to increase with 14% uniaxial strain at 0.3 Hz in both culture systems. Since the ki67 antigen is only detectable for a limited time during cell division (Scholzen and Gerdes 2000), this method might not be as sensitive as the DNA measurements. However, results are well in line with other data that show an increased proliferation rate in comparable stimulation settings (Culbertson et al. 2011; Pingyu et al. 2019; Yang et al. 2004; Zhang and Wang 2010).

In addition, one has to consider that in the spheroid cultures emigrating cells and cells still residing in the spheroid were assessed together, but the proliferation rate and expression profiles could differ due to the differing direct exposition to strain. The effect of the stretching on cells in the different zones of the spheroids can only be hypothesized. Cells emigrated at the bottom of the spheroid culture are directly exposed to the cyclic stretching. The fluid flow around the spheroid cultures might become dynamical during stretching and could influence the outer zones of the spheroids. The nutrition might be facilitated due to movement of the growth media and improved exchange. However, the center of the spheroids is suspected to be rather a rest zone probably barely exposed to the mechanical stimulus.

αSMA is a typical myofibroblastic marker suggesting contractile activity of cells. Myofibroblastic cell activity has been implicated in crimping of the collagen bundles in ligaments (Schwarz et al. 2019; Weiss et al. 2012). A previous study revealed a higher degree of αSMA expression in monolayer cultures of ligamentocytes compared to the in vivo conditions (Schwarz et al. 2019). Detection of an increased αSMA intensity not only in the stretched monolayer but also at the border of the spheroid culture confirmed that the contractile activity of fibroblasts can be increased as response to mechanical stress (Hinz et al. 2001). Additionally, to these intracellular cytoskeletal components, one important factor of the ECM, the sGAGs, were analyzed. A high amount of negatively charged sGAGs is found in native ACL, two to four times higher than in tendons (Duthon et al. 2006). However, neither in the 2D nor in the 3D culture the sGAG content was significantly affected by mechanical stimulation. In agreement with this observation, human scleral fibroblast cultures showed after 48 h of stretching no significant difference in sGAG expression (Shelton and Rada 2007). Considering the fact that based on the DNA measurements, the cell numbers per chamber increased in monolayer and spheroid-derived cultures in response to stretching, the sGAG synthesis per cell might be impaired by strain. In contrast, the most important ECM component in the ACL, collagen type I, responsible for tensile strength (Duthon et al. 2006), was upregulated in spheroid derived cells. This important effect was obviously spheroid specific since we could not find similar data in other studies using monolayer cultures (Kim et al. 2002; Kubo et al. 2020).

DCN is a small leucine-rich proteoglycan and is one of the most present proteoglycans in the ligament (Vogel et al. 1993). DCN plays an important role for integrity and control of the diameter of fibrillary collagen type I (Rühland et al. 2007). The anti-adhesive or adhesion-modulating ECM protein TNC supports the adhesion of cells to fibronectin and it is classified as an “acute phase” protein in response to mechanical stress (Chiquet‐Ehrismann and Chiquet 2003). The upregulated gene expression of TNC in the stretched monolayer and as well as the spheroid culture affirmed this. The increased TNC expression in cells emigrating from spheroids compared to the monolayer might be explained by the fact that it is also involved in cell migration (Midwood and Orend 2009). According to the present study, TNC is also upregulated in rabbit ACL-derived fibroblast spheroids cultured in a dynamic rotatory culture compared to a static spheroid culture as previously shown (Hoyer et al. 2015).

Communication between the tendon-derived fibroblasts and the transport of ions and small signaling molecules is regulated by CXN proteins. The CXN superfamily consists of more than 15 proteins, expressed in a tissue specific manner. Although they all have the same structure, they differ in permeability to intracellular metabolites and second messengers (Cao et al. 1998; Niessen et al. 2000). Cell communication in tendon occurs between extended cell processes of neighbored cells that terminate in CXN32 and 43-positive gap junctions (McNeilly et al. 1996; Waggett et al. 2006). Especially CXN43, prominent in tendons (Waggett et al. 2006), reacts to tensile loading (Banes et al. 1999). Accordingly, it was upregulated in stimulated 2D and 3D culture derived cells presented here.

Tendon or ligament differentiation is mainly regulated by the transcription factors SCX, ERG1 and MKX (Ito et al. 2010; Liu et al. 2017). The correlation between the upregulated cell proliferation and the trend of upregulation of MKX gene expression in the 2D approach is in line with the observation that MKX is involved in effects such as induction of ECM formation in response to mechanosensation in tendon (Kayama et al. 2016). It is also in agreement with the conclusion that uniaxial cyclic stretch induces fibroblast differentiation e.g. by mediating collagen type I synthesis and thereby, supports tendon homeostasis. Tendon/ligament growth and development is also regulated by TNMD, which is a type II transmembrane protein (Docheva et al. 2005; Shukunami et al. 2016). Our results demonstrated that TNMD expression reflected only on a trend of mechanosensitivity in the 2D but not 3D culture derived cells. It has been suggested to enhance cell adhesion (Komiyama 2013). In contrast to(Theiss et al. 2015) who performed static spheroid cultures with equine tenocytes, we could not see any upregulation of the relative gene expression of TNMD in the 3D spheroid-derived fibroblasts. However, in accordance with this observation a previous study (Hoyer et al. 2015) identified a low TNMD expression in spheroid cultures which reflects the low expression in the native mature ACL.

## Limitations

In the present study, a high amplitude of stretching was applied combined with a low frequency (0.3 Hz). This profile did not induce ACL-derived cell dedifferentiation as shown by the expression profile of typical ligament-related components.

However, so far, we did not evaluate possible catabolic responses by including catabolic factors such as MMPs and inflammatory cytokines or prostaglandin E_2_ and cyclooxygenase (COX) 1 and 2 as reported by Wang et al. (2003) which should be analyzed in further experiments. Additionally, we did not check any indicators of transdifferentiation such as towards osteogenesis, since such behavior has been shown for MSCs in long-term cyclic stretching experiments for 14 days (Sumanasinghe et al. 2006).

It has also to be considered when directly comparing the behavior of cells derived from 2D and 3D conditions that the monolayer cultures adhered to pure PDMS, whereas the spheroids in the present study attached to the Geltrex®-coated PDMS chambers. The Geltrex® as well as a 48-h static attachment phase, before the onset of mechanostimulation (in comparison to 24 h in the 2D setting), was necessary to allow stable spheroid attachment. In addition, one should consider the putative effect of Geltrex®, which contains components of the natural basal lamina such as laminin and collagen type IV, not present in larger amounts in the natural ACL.

Looking at the spheroid cultures, gene expression analysis comprised both mRNA transcriptions of non-attached cells in the core of the spheroids together with actively emigrating cells. We can only hypothesize that cells in the inner part of the spheroids are exposed to a reduced mechanical stimulus upon stretching.

## Abbreviations

2D: Two dimensional
3D: Three dimensional
ACL: Anterior cruciate ligament
CLSM: Confocal laser scanning microscope
COL1A1: Gene coding for collagen type I
CXN43: Gene coding for connexin 43
DAPI: 4′,6′-Diamidino-2-phenylindol
DCN: Gene coding for decorin
DMMB: Dimethyl methylene blue
ECM: Extracellular matrix
EDTA: Ethylenediaminetetraacetic acid
ERG1: Early growth response protein
FBS: Fetal bovine serum
FDA: Fluorescein diacetate
GAPDH: Gene coding for glyceraldehyde-3-phosphate dehydrogenase
HBSS: Hank`s balanced salt solution
HUVEC: Human umbilical vein endothelial cells
MKX: Gene coding for Mohawk
PBS: Phosphate-buffered saline
PDMS: Polydimethylsiloxane
PFA: Paraformaldehyde solution
PI: Propidium iodide
rpm: Rounds per minute
RT: Room temperature
SCX: Scleraxis
SD: Standard deviation
sGAG: Sulfated glycosaminoglycan
TBS: Tris-buffered saline
TE: Tris/EDTA
TNC: Gene coding for tenascin C
TNMD: Gene coding for tenomodulin
TRIS: TRIS-(hydoxymethyl)-aminomethane
αSMA: Alpha smooth muscle actin
