# Correction to: Cyclically stretched ACL fibroblasts emigrating from spheroids adapt their cytoskeleton and ligament-related expression profile

**DOI:** 10.1007/s00441-021-03472-1

**Published:** 2021-05-11

**Authors:** Clemens Gögele, Christina Hoffmann, Jens Konrad, Rudolf Merkel, Silke Schwarz, Mersedeh Tohidnezhad, Bernd Hoffmann, Gundula Gesine Schulze-Tanzil

**Affiliations:** 1 Institute of Anatomy and Cell Biology, Paracelsus Medical University, Prof.-Ernst-Nathan Str. 1, 90419 Nuremberg and Salzburg, Nuremberg, Germany; 2 Department of Biosciences, Paris Lodron University Salzburg, Hellbrunnerstr. 34, 5020 Salzburg, Austria; 3 Institute of Biological Information Processing: IBI-2, Forschungszentrum Jülich, 52425 Jülich, Germany; 4 Department of Anatomy and Cell Biology, RWTH Aachen University, Wendlingweg 2, 52074 Aachen, Germany

## Correction to: Cell and Tissue Research https://doi.org/10.1007/s00441-021–03416-9

The published online version contains an error of the figures not caused by the authors were the figures were mixed up in the original publication.

The original article has been corrected.

